# Path Following and Collision Avoidance of a Ribbon-Fin Propelled Underwater Biomimetic Vehicle-Manipulator System

**DOI:** 10.3390/s23167061

**Published:** 2023-08-09

**Authors:** Yanbing He, Xiang Dong, Yu Wang, Shuo Wang

**Affiliations:** 1 School of Electrical Engineering and Automation, Anhui University, Hefei 230601, China; z20201027@stu.ahu.edu.cn; 2 State Key Laboratory of Management and Control for Complex Systems, Institute of Automation, Chinese Academy of Sciences, Beijing 100190, China; yu.wang@ia.ac.cn (Y.W.); shuo.wang@ia.ac.cn (S.W.)

**Keywords:** underwater biomimetic vehicle-manipulator system, nonlinear model predictive control, extended state observers (ESOs), collision avoidance, path following

## Abstract

This paper addresses the problem of path following and dynamic obstacle avoidance for an underwater biomimetic vehicle-manipulator system (UBVMS). Firstly, the general kinematic and dynamic models of underwater vehicles are presented; then, a nonlinear model predictive control (NMPC) scheme is employed to track a reference path and collision avoidance simultaneously. Moreover, to minimize the tracking error and for a higher degree of robustness, improved extended state observers are used to estimate model uncertainties and disturbances to be fed into the NMPC prediction model. On top of this, the proposed method also considers the uncertainty of the state estimator, while combining a priori estimation of the Kalman filter to reasonably predict the position of dynamic obstacles during short periods. Finally, simulations and experimental results are carried out to assess the validity of the proposed method in case of disturbances and constraints.

## 1. Introduction

Over the past few decades, humans have explored and exploited a great number of marine resources; autonomous underwater vehicles (AUVs) have been playing an essential role in underwater operations for marine water quality monitoring as well as oil and gas exploration and other resource surveys [1,2]. With the increasing popularity of underwater operations in the marine environment and the more complex and diverse working environment, the human requirements for underwater robots have further increased, and biomimetic underwater vehicles (BUVs) have attracted a lot of attention from researchers. Through natural selection and continuous self-evolution, fish have evolved near-perfect body mechanisms and control modes of underwater motion. Compared with traditional propeller-equipped underwater robots, BUVs have more maneuverability and can be used to achieve fine attitude adjustment [3,4], especially for low-speed cruising in some particularly restricted areas. In addition, because of the undulating fin movements, they cause less noise and less pollution to the ocean [5].

For the past twenty years, research on the design and motion control of BUVs has received rapid development. Heriot-Watt University’s Sfakiotakis used the parallel bellows actuator as an early undulatory-fin device [6]. Thereafter, Delft University of Technology designed a biomimetic autonomous underwater vehicle with an undulating fin [7]. Niu et al. successfully generated a motion modality by investigating the use of the central pattern generator (CPG) approach for motion learning of an Anguilliform robot fish.

They applied it to a BUV [8]. Wang et al. proposed a backstepping (BP) technique for a bio-inspired robot with undulatory fins to achieve closed-loop depth and course control [9]. Most researchers, however, are more focused on undulatory fin control or some closed-loop path tracking, while BUVs in the actual operation process will be subject to external forces caused by surges, and other interferences and obstacle-avoidance problems are not well-solved.

In recent years, the research commonly has addressed the unknown disturbances of underwater vehicles by designing observers to estimate the unknown disturbances and then compensating for the estimated disturbances by using controllers, which include high-gain observers and an extended state observer (ESO) [10]. In [11], the researchers proposed a motion control system composed of a MIMO PID controller and a high-gain observer, which considers the presence of unmodeled plant dynamics of ROV, measurement errors, noise, and environmental disturbances. In [12], the team proposed a neural extended state observer (ESO) to address the system parameters’ uncertainty and environmental disturbances. In [13], they proposed a linear extended state observer (LESO) applied in the case of sideslip caused by wind, current, and other disturbances in the environment. In addition to the uncertain dynamics and disturbances, another noteworthy problem is the physical constraints for ribbon-fin-propelled underwater vehicles. In recent years, the model predictive control (MPC) algorithm has become an effective optimization method to handle constrained systems [14]. A novel robust MPC method was introduced in [15] to solve the roll constraint for following a straight path. In [16], the team proposed a path-following collision-avoidance MPC method that approximates obstacle shapes as convex polygons and combines the arising norm equality with the dual collision-avoidance inequality constraints.

Most of the above literature for model uncertainty and unknown disturbances in the case of underwater robots’ or surface vessels’ path tracking problems has put forward valid solutions, but few can take into account the obstacle-avoidance problem in the control algorithm, especially in BUVs. In the natural ocean environment, once there is a disturbance, and once deviated from the predetermined trajectory, the risk of collision will greatly increase; underwater robots must safely avoid a collision, and maintain tracking on the predetermined path, which is a tough challenge for the underdriven underwater robot with only surge force and yaw moment. In addition, the control input of an underwater biomimetic vehicle-manipulator system (UBVMS) that relies on fluctuating fins for propulsion is different from that of conventional AUVs and surface vessels, which makes the model uncertainty and the estimation of unknown external disturbances more complex.

In this paper, we propose a concept of integrating an underwater biomimetic vehicle manipulator system (UBVMS) path tracking problem with the multi-object/obstacle-avoidance problem based on the framework of MPC. From a practical perspective, the actual physical platform limitations are incorporated into the constraints of the optimization problem by using the complete nonlinear dynamics constraints of the UBVMS. In experimental tests, since the scan period of the sonar commonly used for underwater obstacle detection is much longer than the sampling period of the NMPC, the speed of obstacle motion is predicted a priori using the Kalman filter. In the case of predicted collisions, not only does it improve the safety of the UBVMS but it also deviates from the defined path as little as possible. To further improve the robustness of obstacle avoidance, the collision cost term in the objective function is shaped using the uncertainty of the state estimation observer. For the consideration of environmental disturbances and the uncertainty of the UBVMS, the total external disturbances, excluding the effects of surge force and yaw moment, are computed using the introduction of extended state observers (ESOs), and the total disturbance term is finally incorporated into the prediction term in NMPC. When the NMPC control cycle ends and the UBVMS reaches a new position, the following optimal input is recalculated based on the current state, and the desired trajectory. Thus, the NMPC algorithm with disturbance compensation, and with obstacle-avoidance constraints incorporated into the control, can be practically applied to the UBVMS even in realistic harsh natural marine environments. Finally, we validate the effectiveness of the algorithm through simulation and experiments with a physical prototype.

## 2. UBVMS Model

### 2.1. Design of the UBVMS

**Figure 1:**
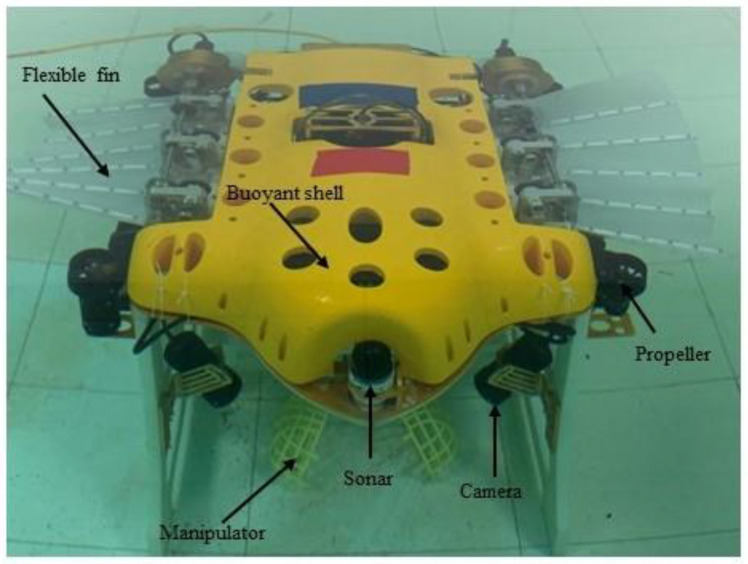
UBVMS.

Bio-inspired long-fin propulsion is introduced into a UVMS (underwater vehicle manipulator system). Compared with the traditional propeller propulsion, it is smaller and can achieve smooth control of the body. The bionic fin propulsors provide excellent stability for underwater operations, and the propeller can provide sufficient power to ensure better maneuverability in the actual water with disturbances. As shown in Figure 1, the UBVMS has two symmetrical long-bionic-fin propulsors, four vertically oriented small propellers, and two flippers at the end of the tail to help improve maneuverability. The UBVMS head is equipped with a depth camera, altimeter, sonar, and many other sensors to help acquire more information about the underwater environment while conducting underwater operations. More details are described in [17]. The physical parameters of UBVMS are in Table 1.

### 2.2. General Kinematics and Dynamics of Underwater Vehicle

The UBVMS has good stability due to its large metacentric height, so it is reasonable to neglect the motion in pitch and roll [9]. With bionic fin propulsors only, the maximum speed of the UBVMS is approximately 0.4 m/s, and the nonlinear damping is negligible at low speeds in the laboratory pool. Based on that, the 3-DOF kinematic and dynamic model can be presented as follows [18]:

(1) $$\dot{x}=\left[\begin{array}{c} J(\varphi )v\\ M^{-1}(-C(\nu )v-Dv+\tau +\tau_{d}) \end{array}\right]=f(x,u)$$

where $x=[\eta^{T}\;v^{T}{]}^{T}\in {\mathbb{R}}^{6}$ is a state vector,$\eta =[x\;y\;\varphi {]}^{T}\in {\mathbb{R}}^{3}$, x(m) and y(m) are the positions, φ(rad) is the heading angle with respect to the earth-fixed frame, and $v={\left[u\;v\;r\right]}^{T}\in {\mathbb{R}}^{3}$ represents the velocities in surge (m/s), sway (m/s), and yaw (rad/s), decomposed in the body-fixed frame. $\tau ={\left[f_{u}\;0\;f_{r}\right]}^{T}$, $f_{u}(N)$, and $f_{r}(N\cdot m)$ are the surge forces and yaw moment produced by the bionic fin propulsors. $\tau_{d}={\left[\tau_{du}\;\tau_{dv}\;\tau_{dr}\right]}^{T}$, $\tau_{du}(N)$, $\tau_{dv}(N)$, and $\tau_{dr}(N\cdot m)$ describe the external forces and moment acting on surge, sway, and yaw, respectively. Based on the previous reasonable assumptions, the system inertial mass matrix (invertible) M and damping matrix D are a diagonal matrix and Coriolis and centrifugal matrix C(v), which are given by:

(2) $$\begin{array}{l} M\overset{\Delta }{=}\left[\begin{array}{ccc} m_{11} & 0 & 0\\ 0 & m_{22} & 0\\ 0 & 0 & m_{33} \end{array}\right],\;D\overset{\Delta }{=}\left[\begin{array}{ccc} d_{11} & 0 & 0\\ 0 & d_{22} & 0\\ 0 & 0 & d_{33} \end{array}\right],\\ \;\;\;\;\;\;\;\;C\overset{\Delta }{=}\left[\begin{array}{ccc} 0 & 0 & -m_{22}v\\ 0 & 0 & m_{11}u\\ m_{22}v & -m_{11}v & 0 \end{array}\right] \end{array}$$

where J(φ)∈SO(3) is the rotation matrix from body-fixed velocities ν to earth-fixed frame velocities $\dot{\eta }$, and has the following structure:

(3) $$J(\varphi )=\left[\begin{array}{ccc} \cos(\varphi ) & -\sin(\varphi ) & 0\\ \sin(\varphi ) & \cos(\varphi ) & 0\\ 0 & 0 & 1 \end{array}\right]$$

The assumptions in this paper are as follows:

1. The UBVMS states x, y, and φ can be measured.
2. The total unknowns $\tau_{du}$, $\tau_{dv}$, and $\tau_{dr}$ are bounded, namely $\left|\tau_{du}\right|\leq \tau_{dumax},\;\left|\tau_{dr}\right|\leq \tau_{drmax},\;\left|\tau_{dv}\right|\leq \tau_{dvmax}$ and $\left|{\dot{\tau }}_{du}\right|\leq \left|{\dot{\tau }}_{dumax}\right|$, $\left|{\dot{\tau }}_{dr}\right|\leq \left|{\dot{\tau }}_{drmax}\right|$, $\left|{\dot{\tau }}_{dv}\right|\leq \left|{\dot{\tau }}_{dvmax}\right|$.

## 3. Problem Formulation

### 3.1. Nonlinear Model Predictive Control

NMPC is a numerical optimization-based control method to achieve the desired optimal performance by systematically ensuring that different constraints’ control is satisfied. The system cost function is constructed, which refers to the error between the state and the desired system state for the next sampling periods, combined with the physical constraints of the UBVMS; then, the cost function is minimized to obtain the optimal control sequence [19,20]. The first element of the control vector sequence is then transformed and fed into the propeller, repeating optimal control at the next sample.

Based on the previously defined system state vectors x and control vectors u, we can obtain the following optimization problem and solve it online:

(4) $$\begin{array}{l} \underset{x(t),u(t)}{\min}J(x,u)=\int_{t=0}^{T}\left\{J_{x}(x(t),x_{ref}(t))+J_{u}(\Delta u(t))+J_{c}(x(t)\right)\}dt\\ s.t.\;\dot{x}=f(x,u),\left(t\right)\in U,\;x(0)=x(t_{0}) \end{array}$$

where f represents system continuous nonlinear equations, $J_{x}$ is a cost function of the error with the desired state $x_{ref}$ of the system, $J_{u}$ and $J_{c}$ control input and collision cost penalties, respectively, and U is a set of feasible control vector inputs. Since the NMPC is to be used with a practical system, the thrust of the bionic fin propulsor is limited, and such hard constraint must be considered at every control cycle. The input control vector constraints are as follows:

(5) $$U=\left\{u\in {\mathbb{R}}^{3}|\left[\begin{array}{c} \begin{array}{c} f_{u\min}\\ f_{v\min} \end{array}\\ f_{r\min} \end{array}\right]\leq u\leq \left[\begin{array}{c} \begin{array}{c} f_{u\max}\\ f_{v\max} \end{array}\\ f_{r\max} \end{array}\right]\right\}$$

It is worth noting here that the UBVMS is an underdriven underwater vehicle and, in fact, $f_{v\min}=f_{v\max}=0$. Now, for a detailed description of the cost function part in Equation (4), the first term of the cost function $J_{x}(x(t),\;x_{ref}(t))$ represents the state cost, which penalizes deviating from the desired state $x_{ref}$; the details are as follows:

(6) $$J_{x}(x(t),x_{ref}(t))=||x(t)-x_{ref}(t)|{|}_{Q_{x}}^{2}$$

where $Q_{x}\in {\mathbb{R}}^{6\times 6}$ represents positive definite weight matrices. The second term of the cost function $J_{u}(\Delta u(t))$ is to ensure the smoothness of the system control vector throughout the control process u, related to the previous control action; the details are as follows:

(7) $$J_{u}(\Delta u(t))=\;||\;u(t+T)-u(t){\;||}_{Q_{u}}^{2}\;$$

**Figure 2:**
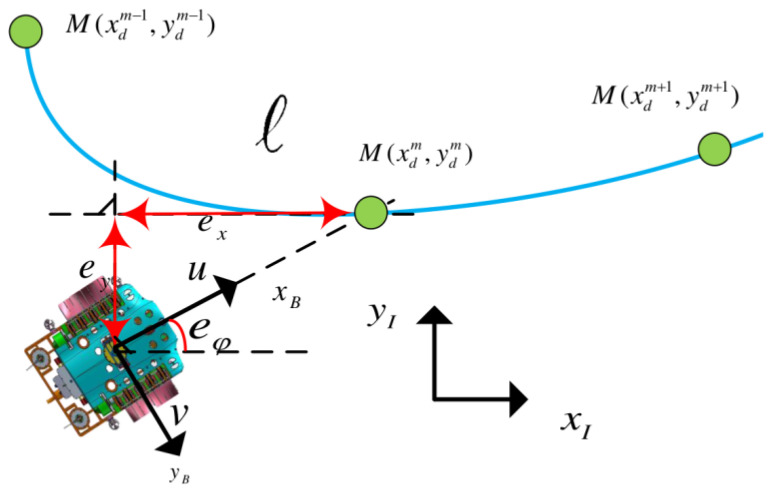
The global guidance principle.

The last term of the cost function is to expect the UBVMS to have a sufficiently safe distance from dynamic obstacles when working underwater, and we introduce a collision cost term similar to the logistic function, which ensures that the cost function is continuously differentiable. Formally,

(8) $$J_{c}(x(t))=\sum_{i=1}^{n}\frac{Q_{c,i}}{1+\exp k_{i}(d_{i}(t)-r_{th,i}(t))}$$

where $Q_{c,i}$ is a design weight, $d_{i}(t)$ is the Euclidean distance from the i-th dynamic obstacle, and $k_{i}>0$ is a positive constant that denotes the smoothness of the obstacle avoidance trajectory. As the value of $k_{i}$ increases, the cost function becomes very sharp, which can make the optimization problem more challenging to solve, so the value of $k_{i}$ can be chosen to be adjusted according to different environmental situations [21]. The global reference path is provided by the global planner; we use a cubic spline to interpolate, calculate the reference yaw and divide the whole reference trajectory into segments of the same size; a sequence of path segments connecting path points M forms a global reference path l. The UBVMS chooses the closest point as the tracking target, as shown in Figure 2.

### 3.2. Uncertainty Consideration and Disturbance Estimation

For the design of a UBVMS control system, system uncertainty is a fundamental issue that cannot be avoided, coming from the internal (structure or parameters) elements of the system on the one hand and the external (environmental disturbances) elements on the other [22]. NMPC is a model-based controller, and it is essential to improve the model’s accuracy as much as possible, so before applying it to practical objects, we need to consider the impact on the control quality due to the model’s internal uncertainty and external environmental disturbances. In addition, NMPC is also based on the model to make future state predictions. Then, in the optimization based on the prediction, if the disturbance is not compensated for in time, the control effect will become terrible, and even the system will become unstable. We add disturbance terms to the prediction model of NMPC, such as $\tau_{du}$, $\tau_{dr}$ and $\tau_{dv}$ shown in Equation (1). The disturbance observer will compute the disturbance in each control cycle. When operating in a complex marine environment, the operational performance of marine vehicles is susceptible to external environmental disturbances. The disturbance observers are widely applied in various marine applications and have a practical significance [23]. In [22], the estimation ability of ESO for uncertainty is discussed and analyzed, and the researchers show that ESO can deal with a large number of disturbances. ESO is the core of ADRC proposed by [24], and then Gao proposed an improved linear ADRC control algorithm, LADRC, based on the classical ADRC idea [25]. In [26], LESO is introduced to estimate the uncertain parameters and external disturbances, and the surge velocity is calculated.

In our work, ESO is used to estimate the velocity and external disturbances in the motion coordinate system, and the nonlinear function in ESO is modified by introducing a hyperbolic tangent function to replace the original nonlinear function, which is a bounded singular function with more adjustable parameters than the nonlinear function and can be used to flexibly adjust the limits of the error magnitude as follows:

(9) $$\beta \tanh(\lambda z)=\frac{e^{\lambda z}-e^{-\lambda z}}{e^{\lambda z}+e^{-\lambda z}}$$

λ and β are positive parameters that can be used to adjust the limit range of the amplitude and the rate of change in the amplitude, respectively. According to the UBVMS dynamic model (see Equation (1)), the disturbance moment observer of the external environment in the yaw direction can be:

(10) $$\left\{\begin{array}{l} \dot{\hat{\varphi }}=\hat{r}-l_{0}\tanh(\lambda_{1}(\hat{\varphi }-\varphi ))\\ \dot{\hat{r}}={\hat{\tau }}_{dr}-l_{1}\tanh(\lambda_{1}(\hat{\varphi }-\varphi ))+\frac{\left(m_{11}-m_{22}\right)}{m_{33}}uv-\frac{d_{33}}{m_{33}}r+b_{0}\frac{f_{d}}{m_{33}}\;\\ {\dot{\hat{\tau }}}_{dr}=-l_{2}\tanh(\lambda_{1}(\hat{\varphi }-\varphi )) \end{array}\right.$$

where $l_{0}$, $l_{1}$, and $l_{2}$ are positive parameters, which could adjust the feedback range of estimation errors. Increasing their values within a certain range can improve the estimation accuracy, but it also brings oscillations as a result [27]. $\hat{r}$, ${\hat{\tau }}_{dr}$, and $\hat{\varphi }$ represent the respective estimated values, and $b_{0}$ is the compensation factor.

(11) $$\left\{\begin{array}{l} \dot{\hat{\zeta }}=\hat{u}-l_{3}\tanh(\lambda_{2}(\hat{\zeta }-\zeta ))\\ \dot{\hat{u}}={\hat{\tau }}_{du}-l_{4}\tanh(\lambda_{2}(\hat{\zeta }-\zeta ))+\frac{m_{22}}{m_{11}}vr-\frac{d_{11}}{m_{11}}u+b_{1}\frac{f_{u}}{m_{11}}\;\\ {\dot{\hat{\tau }}}_{du}=-l_{5}\tanh(\lambda_{2}(\hat{\zeta }-\zeta )) \end{array}\right.$$

(12) $$\left\{\begin{array}{l} \dot{\hat{\chi }}=\hat{v}-l_{6}\tanh(\lambda_{3}(\hat{\chi }-\chi ))\\ \dot{\hat{v}}=\hat{d_{v}}-l_{7}\tanh(\lambda_{3}(\hat{\chi }-\chi ))-\frac{m_{11}}{m_{22}}ur-\frac{d_{22}}{m_{22}}v\\ {\dot{\hat{\tau }}}_{dv}=-l_{8}\tanh(\lambda_{3}(\hat{\chi }-\chi )) \end{array}\right.$$

**Figure 3:**
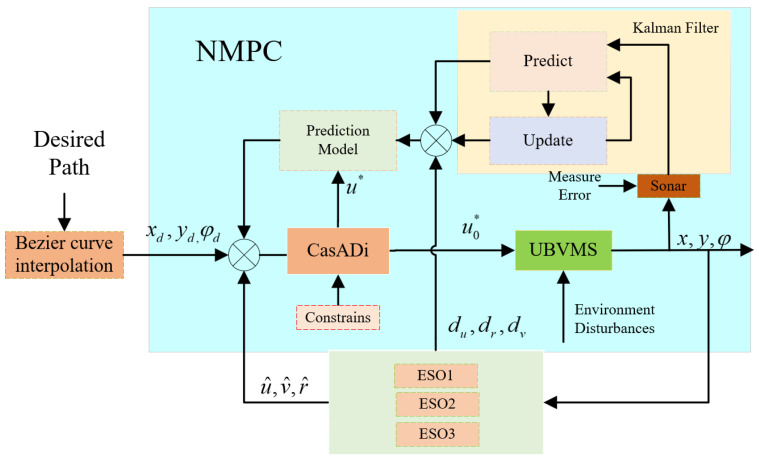
Diagram of the proposed control scheme.

ζ=u⋅t and χ=v⋅t denote the displacement of the surge velocity and sway velocity in the body-fixed frame, respectively, where $l_{3}$,…, $l_{8}$ are positive parameters, $\hat{u},\;{\hat{\tau }}_{du},\;\hat{\zeta }$, $\hat{v}$, ${\hat{\tau }}_{dv}$, and $\hat{\chi }$ represent the respective estimated values, and $b_{1}$ is the compensation factor, $u^{*}$ is the optimal control input for the solution. Notably, the estimates for the disturbance terms are added to the prediction module of the controller NMPC and updated by ESOs at each sampling cycle. There is a more intuitive representation in Figure 3 and Algorithm 1.
**Algorithm 1**: NMPC for UBVMS Algorithm1: Initialize system parameters;2: **Repeat**;3:     Measure and compute estimated velocities, along with system state $x(t_{0})$ disturbance term updates from ESOs;4:    Compute future predicted states $\hat{x}(t+nT_{s}),n=1,\ldots ,N$ and predicted obstacle states (17);5:     Solve the OCP (4) and obtain the desired optimal solution sequence $u^{*}(t+nT_{s}),n=1,\ldots ,N-1$;6:    Allocate the desired optimal solution $u_{0}^{*}$ and convert to bionic fin propulsor;7:    **If** sonar scan completed, **then**8:          Kalman filter update: compute $r_{th,k}(t)$ and update $P_{t}^{-}$, $P_{t}$;9:     **Else** $P_{t}^{-}=P_{t}^{-}$, $P_{t}=P_{t}$;10:    **End if**11:   Compute the desired reference path and save system data;12:   Update: nmpciter=nmpciter+1;13: **Until** stop.

### 3.3. Robust Collision Avoidance

In the previous section, we discussed the uncertainty in the UBVMS; in order to further improve the robustness of control in underwater environments, the uncertainty of state estimation of dynamic obstacles in water is considered, inspired by the literature [28]. With non-negligible uncertainty in predicting obstacle states due to the inaccuracy of the prediction model and the interference of external environmental measurement noise, we should be more conservative and allow the system to approach the obstacle by increasing $r_{th,i}(t)$ along the prediction horizon. In the natural marine underwater environment, dynamic obstacles are hardly ever in actual uniform motion, so to simulate the trajectory of dynamic obstacles closer to the real situation, we chose the uniform motion mode with Gaussian noise interference as follows:

(13) $$p_{i}^{obs}(t)=p_{i}^{obs}(t_{0})+(v_{i}(t_{0})+w_{0})(t-t_{0})\;$$

where $w_{0}$ is a random number $w_{0}(t)\;\sim N(0,Q_{0}(t))$, and $v_{i}(t_{0})$ and $p_{i}^{obs}(t_{0})$ are the velocity and position at the current moment, respectively. To obtain an accurate estimation of the obstacle state and compute the uncertainty in the state estimator of objects in the process, for the proposed obstacle motion model (12), we choose to perform uncertainty propagation based on a Kalman filter (KF) [28]. The KF can be formulated as:

Predict:

(14) $$\left\{\begin{array}{l} {\hat{x}}_{t}^{-}=F{\hat{x}}_{t-1}\\ P_{t}^{-}=FP_{t-1}F^{T}+Q_{t} \end{array}\right.$$

Update:

(15) $$\left\{\begin{array}{l} K_{t}=P_{t}^{-}H^{T}{\left(HP_{t-1}H^{T}+R_{t}\right)}^{-1}\\ {\hat{x}}_{t}={\hat{x}}_{t}^{-}+K_{t}(Z_{t}-H{\hat{x}}_{t}^{-})\;\\ P_{t}=(I-K_{t}H)\;P_{t}^{-}\; \end{array}\right.$$

where $x_{t}={\left[x^{obs},y^{obs},v^{obs}\right]}^{T}$ is a state vector, $P_{t}^{-}$ and $P_{t}$ represent the a priori (predicted) and a posteriori state vectors, respectively, and $Z_{t}={\left[x^{o},y^{o},v^{o}\right]}^{T}$ is the measurement vector acquired by employing sonar at moment t. F is the state transition Jacobian matrix, and H is the observation matrix. $Q_{t}$ is the covariance matrix of the motion obstacle system noise, and $R_{t}$ is the covariance matrix of the observation noise, and we must note that all of these are positive parameters. The reasonable selection of $Q_{t}$ and $R_{t}$ is crucial for the development of the Kalman filter [29]. We express the uncertainty of the i−th dynamic obstacle at moment t by solving the equation $P_{t}^{-}=FP_{t-1}F^{T}+Q_{t}\;$ with maximum eigenvalue $\delta_{k}(t)$. $r_{th,i}(t)$ can be calculated using the following equation:

(16) $$r_{th,i}(t)=r_{th}+10\delta_{i}(t)$$

where $r_{th}$ represents constant parameters, and the use of an enclosing sphere to approximate the uncertainty ellipsoid makes the bound more conservative [28]. Compared to the travel space of a mobile robot on land, the moveable space of a UBVMS is ample, and it is wiser to convert the complex elliptical distance calculation to a circular distance calculation in terms of computational complexity. In addition, since usually the sonar scanning time to ascertain the obstacle position is relatively long, the time to obtain the updated obstacle position is much longer than the NMPC sampling period. Since we cannot determine the latest state of the obstacle movement in every control cycle, to carry out a safer motion control, reasonable use of the Kalman filter a priori information for obstacle position prediction is critical. The obstacle trajectories are predicted as follows:

(17) $$p_{obs\;i}^{pre}(t_{0}+n\cdot T_{s})=p_{obs\;}{}_{i}(t_{0})+n\cdot T_{s}\cdot v^{o-}(t_{0})\;,\;n\in (1,\ldots ,N)$$

where $P_{obs\;i}(t_{0})$ denotes the obstacle position at moment $t_{0}$, $T_{s}$ is the NMPC sampling period, and $v{\left(t_{0}\right)}^{o-}$ denotes the i−th obstacle prior velocity obtained by Kalman filtering at moment $t_{0}$. In the NMPC prediction module, along with the number of prediction steps n from 1 to N, the obstacle prediction position is also updated until the Kalman filter is updated for the prior velocity. The proposed NMPC algorithm is briefly described in Algorithm 1.

The control structure of the proposed system is schematically shown in Figure 3. CasADi is an open-source software framework for numerical optimization that is well suited to nonlinear optimization problems, especially those constrained by differential equations [30,31]. In the simulation and experiment part of this paper, CasADi solver plugin ipopt is selected to solve the optimal control problem.

## 4. Simulations and Experimental Results

### 4.1. Simulation Results

The physical parameters for the UBVMS are chosen as [9]: $m_{11}=57.5$, $m_{22}=61.3$, $m_{33}=1.15$, $d_{11}=53$, $d_{22}=58$, and $d_{33}=3.1$. The initial value of the system state vector is 0. The NMPC-related control parameters are as follows: $T_{s}=0.2\;s$, $N_{p}=25$, $N_{c}=1$, and weighting matrices $Q_{x}=diag\{55.0,\;55.0,\;5.0,\;1.0,\;800,\;5.0\}$ and $Q_{u}=\;\mathrm{diag}\{10.0,\;25.0\}$. The collision penalty weights and prediction penalty weights are $Q_{c}=20$, $Q_{cp}=10$. The desired surge velocity in the body-fixed frame is 0.2 m/s, and $l_{1}=3.6$, $l_{2}=4.32$, $l_{3}=1.728$, $l_{4}=2.4$, $l_{5}=1.92$, $l_{6}=1.516$, $l_{7}=2.4$, $l_{8}=1.92$, and $l_{9}=0.528$. The external disturbance is $\tau_{du}=0.5\sin(0.75\times 0.1t)$, $\tau_{dv}=0.4\sin(0.75\times 0.1t)$, $\tau_{dr}=0.3\sin(0.75\times 0.12t)$.

Dynamic obstacles are initially positioned as follows: $\left(x_{t_{0}}^{obs},y_{t_{0}}^{obs})=(6,3\right)$. The radius of the UBVMS in the simulation is 0.3 m, and the radius of dynamic obstacle expansion is 0.5 m. The initial velocity of the obstacle is −0.15 m/s, as well as adding to the Gaussian distribution N(0, 0.02). In the multi-obstacle simulation, the initial positions of the three dynamic obstacles are as follows: (5.4, 4.0), (4.15, 1.0), (5.0, 6.0). To better verify the effectiveness of the proposed method, the three dynamic obstacles make a linear motion with initial velocity −0.15 m/s and an additional Gaussian distribution N(0, 0.02) at the approach of the UBVMS.

**Figure 4:**
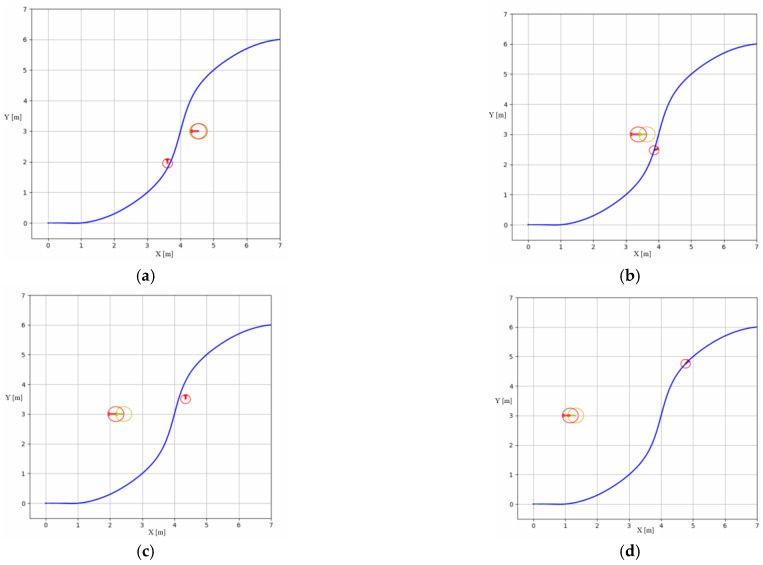
Process snapshots. (**a**) t = 35.02 s. (**b**) t = 37.20 s. (**c**) t = 46.00 s. (**d**) t = 52.6 s.

Figure 4 shows the more critical moments of the BUMVS in the path tracking process. The blue curve is the desired path, the small red circle is the UBVMS, the large one is the dynamic obstacle, and the marker symbol above the red circle indicates the yaw angle at this moment. The yellow circle indicates the posterior position based on the Kalman filter. In Figure 4a, it can be seen that because there is an obstacle in front of the right of the UBVMS at this moment, it chooses to deviate from the intended trajectory to the left to avoid a collision; notably, in Figure 4b, when the obstacle trajectory is predicted by combining a priori messages in the KF, the controller chooses to drive to the right with lower probability of collision. Figure 4c,d show that the UBVMS returns to the original desired path soon after avoiding this collision.

**Figure 5:**
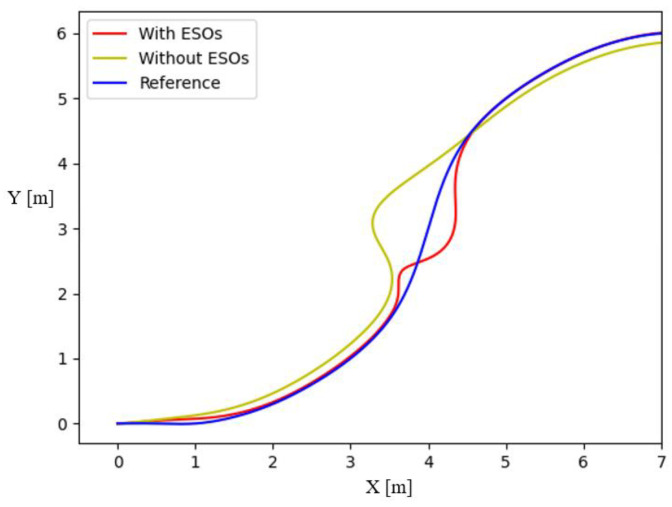
The curve path tracking.

**Figure 6:**
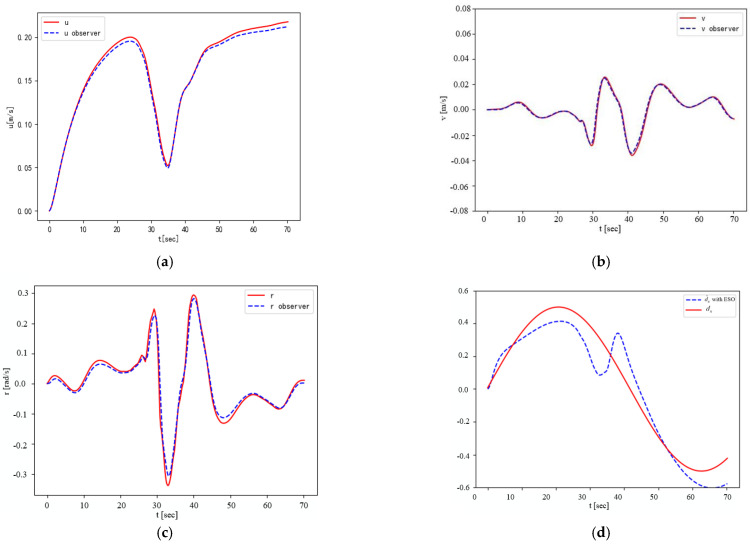

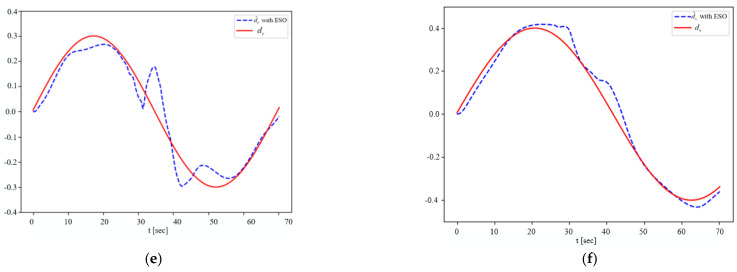
Estimation of velocity and disturbance. (**a**) Surge velocity. (**b**) Sway velocity. (**c**) Yaw velocity. (**d**) ${\hat{d}}_{u}(t)$. (**e**) ${\hat{d}}_{r}(t)$. (**f**) ${\hat{d}}_{v}(t)$.

**Figure 7:**
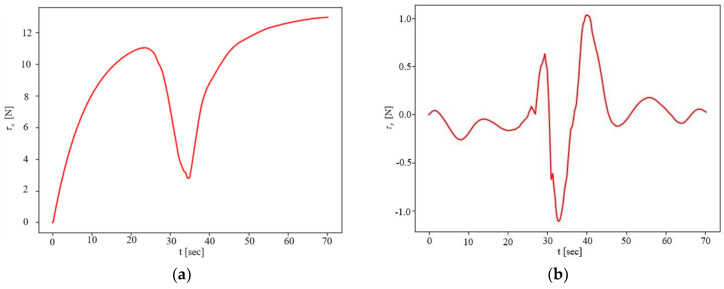
Surge force and yaw moment. (**a**) Surge force. (**b**) Yaw moment.

In Figure 5, note that there is always obvious error in path tracking without the ESO disturbance observer, while the NMPC with disturbance observation compensation can achieve error-free tracking. In addition, it can be seen that when the UBVMS has access to the predicted future trajectory of the obstacle, it chooses the opposite direction of motion to the obstacle (positive *x*-axis direction) to avoid collisions. The surge, sway, and yaw velocities are presented in Figure 6a–c, respectively. Here, it is interesting to note that the surge velocity quickly reaches the desired speed of 0.2 m/s after avoiding the obstacle, and sway velocity is also maintained at approximately 0 m/s. Figure 6d–f show the ability of ESOs to estimate time-varying disturbances in the external environment; although the input control vector forces and moments cause some oscillations during sharp changes, the disturbances can still be approximately calculated in a short time. Figure 7a and 7b illustrate the surge thrust and yaw moment computed by the CasADi solver, respectively. When constraining the variation in the control vector Δu in the objective function, the control vector is smoother, and the effect is obvious for the yaw moment.

**Figure 8:**
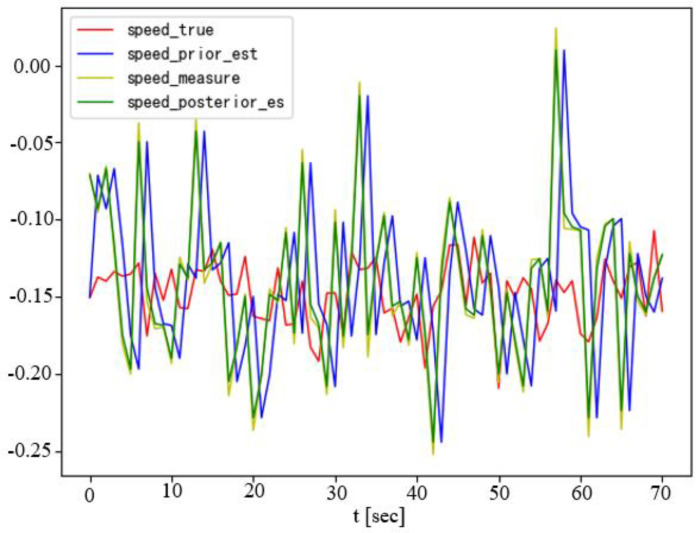
Obstacle velocity produced by KF estimator.

Figure 8 shows the results of the Kalman filter estimating and predicting the velocity of an obstacle with a non-uniform motion. In the same scenario as Figure 4 above, we add multiple dynamic obstacles near the predetermined desired trajectory of the UBVMS and, for each added obstacle, we carry out ten consecutive simulation experiments. The results of the scenario experiments are shown in Table 2. 

The simulation results show that when a priori prediction is added to NMPC, the situation is much better than without it. First, we have fewer collisions, although we still fail four times with three dynamic obstacles added; this is probably more related to the maneuverability of the UBVMS itself, which has better stability but does not move very fast. Second, it maintains a sufficient safety distance, and in the case of only one obstacle, it not only has a 100% success rate but also has a shorter driving distance.

### 4.2. Experimental Results

To further verify the performance of the proposed algorithm, we conducted experiments on the UBVMS in an indoor pool. The position and yaw angle of the UBVMS (x,y,φ) were obtained by the global camera located above the pool, the data were transmitted via wired fiber optic cable, the velocity was calculated using an ESO differential tracker, and the sonar acquired the position of the obstacle.

**Figure 9:**
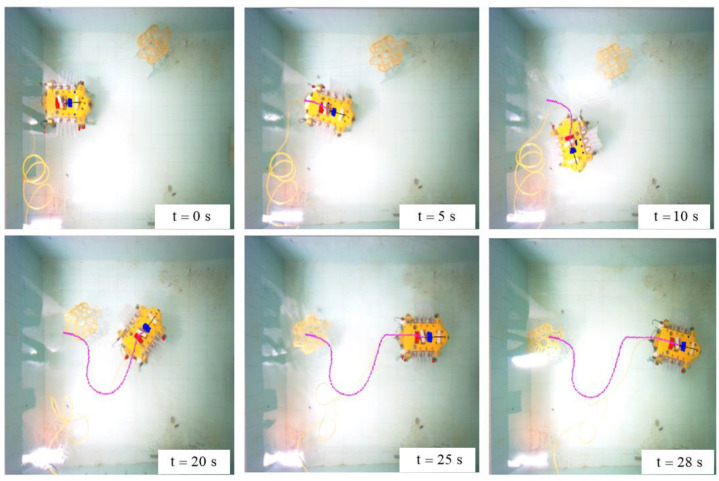
Screenshot sequence of the complete experiment.

Figure 9 shows the process of the UBVMS performing linear tracking and avoiding moving obstacles. It can be observed that the proposed control system can enable the UBVMS to smoothly perform path tracking as well as prevent a collision. When the sonar detects the obstacle at a close distance, the surge of collision cost of the objective function drives the UBVMS to deviate from the desired trajectory to avoid the collision, and then it returns to the desired path largely without overshoot and achieves zero error before reaching the target end point. The starting position of the UBVMS is the origin in geodesic coordinates, the initial velocity is 0, and the tracking path is a straight line of length 2 m.

**Figure 10:**
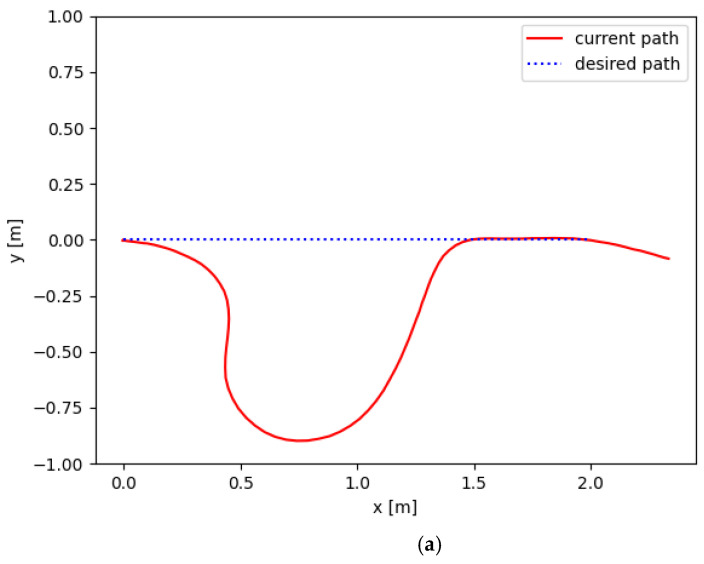

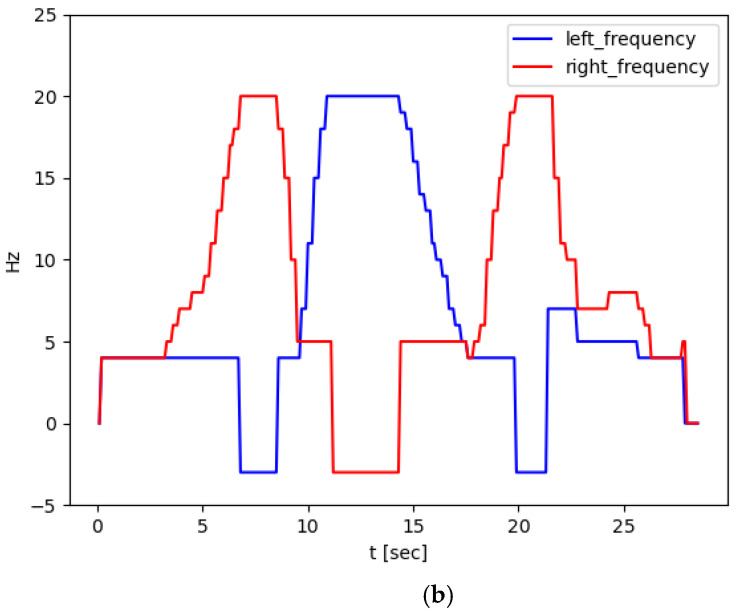
Experimental results. (**a**) Desired path and current path. (**b**) Control frequency of bilateral fins.

It is noteworthy that due to the relatively small size of the indoor swimming pool, when the UBVMS moves at the maximum frequency (20 Hz), the additional interference caused by the reflected wave in the swimming pool cannot be ignored. Therefore, when reaching the end of the desired path, the UBVMS still has a slight trajectory offset due to inertia and disturbance. The calculated $\tau ={\left[f_{u}\;0\;f_{r}\right]}^{T}$ is multiplied by the respective scale factor to complete the mapping from force and torque to bilateral fin frequency. Figure 10b shows the time evolution of the control frequency of bilateral fins.

## 5. Conclusions

In this article, we have investigated the path following and dynamic obstacle avoidance of the UBVMS propelled by undulatory ribbon-fins with model uncertainties, external environmental disturbances, and input constraints. To achieve minimized path tracking error and robust obstacle avoidance, a general kinematic/dynamic model of the underwater vehicle was developed; external forces were modeled as unmeasured disturbances and were estimated using three ESOs fed into the NMPC prediction model. Moreover, we considered the uncertainty of the state estimator, combining the a priori estimation of the Kalman filter to predict the position of dynamic obstacles. The simulation results show that the proposed method can track the curved trajectory and achieve multi-obstacle avoidance successfully in the event of time-varying disturbances. Our experiments showed that this path-following and collision-avoidance approach for the UBVMS is valid and maintains system stability.

## Figures and Tables

**Table 1:** Physical parameters of UBVMS.

Parameter	Value	Parameter	Value
Mass	55 kg	Buoyancy	54.3 kg
Manipulator mass	2.5 kg	Fin length	0.58 m
Length	0.95 m	Maximum frequency	20 Hz
Width	0.7 m	Maximum linear velocity	0.43 m
Height	0.6 m	Maximum steering speed	0.89 rad/s

**Table 2:** Multi-obstacle simulation results.

Number of Obstacles	Without Priori (Predicted)			With Priori (Predicted)		
	Clearance Mean (m)	Traveled Distance Mean (m)	%Failures (Collisions)	Clearance Mean (m)	Traveled Distance Mean (m)	%Failures (Collisions)
1	0.0487	11.6114	60%	**0.3100**	**11.4018**	**100%**
2	0.0172	**10.7272**	20%	**0.3245**	10.8660	**90%**
3	0.0574	**10.8160**	20%	**0.3295**	11.3869	**60%**

## Data Availability

Not applicable.

## Abbreviations

The following abbreviations are used in this manuscript:

UBVMS	underwater biomimetic vehicle-manipulator system
AUV	autonomous underwater vehicles
BUV	biomimetic underwater vehicles
CPG	central pattern generator
NMPC	nonlinear model predictive control
ESO	Extended state observers
KF	Kalman Filter

## References

[1] Wu Z., Liu J., Yu J., Fang H. (2017). Development of a novel robotic dolphin and its application to water quality monitoring. IEEE/ASME Trans. Mechatron..

[2] Richmond K., Flesher C., Lindzey L., Tanner N., Stone W.C. Sunfish^®^: A human-portable exploration auv for complex 3d environments. Proceedings of the OCEANS 2018 MTS/IEEE Charleston.

[3] Lauder G.V., Anderson E.J., Tangorra J., Madden P.G. (2007). Fish biorobotics: Kinematics and hydrodynamics of self-propulsion. J. Exp. Biol..

[4] Neveln I.D., Bai Y., Snyder J.B., Solberg J.R., Curet O.M., Lynch K.M., MacIver M.A. (2013). Biomimetic and bio-inspired robotics in electric fish research. J. Exp. Biol..

[5] Zhou C., Low K.H. (2011). Design and locomotion control of a biomimetic underwater vehicle with fin propulsion. IEEE/ASME Trans. Mechatron..

[6] Sfakiotakis M., Laue D.M., Davies B.C. An experimental undulating-fin device using the parallel bellows actuator. Proceedings of the 2001 ICRA, IEEE International Conference on Robotics and Automation (Cat. No. 01CH37164).

[7] Vercruyssen T.G.A. (2010). Phase Resolved PIV Analysis of an Undulating Fin: Experimental Investigation of the Galatea Propulsion Mechanism. Master’s Thesis.

[8] Niu X., Xu J., Ren Q., Wang Q. (2013). Locomotion learning for an anguilliform robotic fish using central pattern generator approach. IEEE Trans. Ind. Electron..

[9] Wang R., Wang S., Wang Y., Tan M., Yu J. (2017). A paradigm for path following control of a ribbon-fin propelled biomimetic underwater vehicle. IEEE Trans. Syst. Man Cybern. Syst..

[10] Cui R., Chen L., Yang C., Chen M. (2017). Extended state observer-based integral sliding mode control for an underwater robot with unknown disturbances and uncertain nonlinearities. IEEE Trans. Ind. Electron..

[11] Fernandes D.D.A., Sørensen A.J., Pettersen K.Y., Donha D.C. (2015). Output feedback motion control system for observation class ROVs based on a high-gain state observer: Theoretical and experimental result. Control. Eng. Pract..

[12] Wan L., Zeng J., Li Y., Qin H., Zhang L., Wang J. (2019). Neural observer-based path following control for underactuated unmanned surface vessels with input saturation and time-varying disturbance. Int. J. Adv. Robot. Syst..

[13] Liu L., Wang D., Peng Z., Li T., Chen C.P. (2018). Cooperative path following ring-networked under-actuated autonomous surface vehicles: Algorithms and experimental results. IEEE Trans. Cybern..

[14] Lee J., Chang H.J. (2018). Analysis of explicit model predictive control for path-following control. PLoS ONE.

[15] Li G., Zhang J., Liu Z., Wang L., Sun T. Predictive control for straight path following of underactuated surface vessels with roll constraints. Proceedings of the 2016 Chinese Control and Decision Conference (CCDC).

[16] Helling S., Roduner C., Meurer T. On the dual implementation of collision-avoidance constraints in path-following MPC for underactuated surface vessels. Proceedings of the 2021 American Control Conference (ACC).

[17] Wang Y., Wang R., Wang S., Tan M., Yu J. (2019). Underwater bioinspired propulsion: From inspection to manipulation. IEEE Trans. Ind. Electron..

[18] Fossen T.I. (1999). Guidance and Control of Ocean Vehicles. Ph.D. Thesis.

[19] Zhang Y., Liu X., Luo M., Yang C. (2019). MPC-based 3-D trajectory tracking for an autonomous underwater vehicle with constraints in complex ocean environments. Ocean. Eng..

[20] Long C., Qin X., Bian Y., Hu M. (2021). Trajectory tracking control of ROVs considering external disturbances and measurement noises using ESKF-based MPC. Ocean. Eng..

[21] Zhao C., Wang D., Hu J., Pan Q. (2021). Nonlinear model predictive control-based guidance algorithm for quadrotor trajectory tracking with obstacle avoidance. J. Syst. Sci. Complex..

[22] Yang X., Huang Y. Capabilities of extended state observer for estimating uncertainties. Proceedings of the 2009 American Control Conference.

[23] Gu N., Wang D., Peng Z., Wang J., Han Q.L. (2022). Disturbance observers and extended state observers for marine vehicles: A survey. Control. Eng. Pract..

[24] Han J. (2009). From PID to active disturbance rejection control. IEEE Trans. Ind. Electron..

[25] Gao Z. Scaling and bandwidth-parameterization based controller tuning. Proceedings of the 2003 American Control Conference.

[26] Li Z., Li R., Bu R. (2021). Path following of under-actuated ships based on model predictive control with state observer. J. Mar. Sci. Technol..

[27] Li Z., Bu R. (2021). Trajectory tracking of under-actuated ships based on optimal sliding mode control with state observer. Ocean. Eng..

[28] Kamel M., Alonso-Mora J., Siegwart R., Nieto J. Robust collision avoidance for multiple micro aerial vehicles using nonlinear model predictive control. Proceedings of the 2017 IEEE/RSJ International Conference on Intelligent Robots and Systems (IROS).

[29] Mousazadeh H., Jafarbiglu H., Abdolmaleki H., Omrani E., Monhaseri F., Abdollahzadeh M.R., Mohammadi-Aghdam A., Kiapei A., Salmani-Zakaria Y., Makhsoos A. (2018). Developing a navigation, guidance and obstacle avoidance algorithm for an Unmanned Surface Vehicle (USV) by algorithms fusion. Ocean. Eng..

[30] Rao A.V. (2009). A survey of numerical methods for optimal control. Adv. Astronaut. Sci..

[31] Andersson J.A., Gillis J., Horn G., Rawlings J.B., Diehl M. (2019). CasADi: A software framework for nonlinear optimization and optimal control. Math. Program. Comput..

